# The Anti-Depressive Effects of Hesperidin and the Relative Mechanisms Based on the NLRP3 Inflammatory Signaling Pathway

**DOI:** 10.3389/fphar.2020.01251

**Published:** 2020-08-14

**Authors:** Lulu Xie, Zhimin Gu, Haizhao Liu, Beitian Jia, Yiyang Wang, Min Cao, Ruiwen Song, Zhaiyi Zhang, Yuhong Bian

**Affiliations:** ^1^School of Intergrative Medicine, Tianjin University of Traditional Chinese Medicine, Tianjin, China; ^2^School of Management, Tianjin University of Traditional Chinese Medicine, Tianjin, China

**Keywords:** depression, inflammation, microglia, NLRP3, hesperidin

## Abstract

There is increasing evidence showing that inflammation is associated with depression in humans. Hesperidin, a natural bioflavonoid, has performed excellent effects on depression. The aim of this research was to investigate the therapeutic eﬀect of hesperidin on chronic unpredictable mild stress (CUMS)-induced rats. The sucrose preference test (SPT), forced swimming test (FST), and open ﬁeld test (OFT) were performed to measure the depression-related symptoms. The enzyme-linked immunosorbent assay (ELISA) was used to determine the concentrations of interleukin (IL)-1β, IL-6, and tumor necrosis factor (TNF)-α in the prefrontal cortex (PFC) of rats and cellular supernatant. PCR and Western blot were used to monitor the differences of NLRP3, caspase-1, ASC activation in the levels of genes and proteins in the PFC of rats and microglia. The activation of microglia was determined using immunofluorescence staining and flow cytometry assay. Our results show that hesperidin treatment signiﬁcantly relieved depressive like behaviors in CUMS rats. In addition, hesperidin decreased the expression levels of IL-1β, IL-6, TNF-α, NLRP3, caspase-1, and ASC in the PFC and microglia. This study investigated that hesperidin treatment ameliorated CUMS-induced depression by suppressing microglia and inflammation.

## Introduction

Depression is a common neuropsychiatric disorder, characterized by significant and lasting negative emotions, suicidal thoughts, loss of appetite, sleep disorders, down-regulated energy, and other somatic symptoms. In the recent WHO reports, more than 300 million people (4.4%) suffer from depression globally, so it could be a guiding factor of disease burden by 2030 (Shrivastava et al., 2017; Iniguez et al., 2018). Recently, an increasing number of studies have shown that depression and anxiety disorders might be caused by early-life inflammation in adulthood. High levels of neuro-inflammatory cytokines such as interleukin (IL)-1β, tumor necrosis factor-α (TNF-α), and IL-6 have been identified in patients with depression. Koo et al. suggested that the depression-related symptoms which were induced by acute and chronic stress were mediated by IL-1β in 2008 (Koo and Duman, 2008). Microglia was proposed as the main immune cells in the central nervous system (CNS) (Xu et al., 2016) and it has been shown that depression is in connection with the dynamic changes of function and structure of microglia (Kreisel et al., 2014; Tan et al., 2017). Glial cells (including astrocyte and microglial cells) is a well-known and major source of CNS inflammatory cytokines (Ransohoff and Brown, 2012). As the main effector cells of the nervous system, microglia can produce IL-lβ which promotes the occurrence and development of inflammation which then leads to depression (Mason et al., 2001; Xu et al., 2016). It has been reported that the stress-sensitive NLRP3 inflammasome, which is activated in blood cells from depression patients, plays an important role in priming of microglia, such as induced the activation and amplified expression of IL-1β in the CNS. (Herman and Maria, 2018). Further studies have shown that the density of microglial cells is elevated in the prefrontal cortex (PFC) of patients with depression. The NLRP3 inflammasome mediates the maturation and secretion of IL-1β and serves as an upstream regulator in the expression of the IL-1β (Cassel et al., 2009; Lamkanfi and Kanneganti, 2010; Su et al., 2017). Also, the activation of microglial NLRP3 inflammasome may mediate the expression of IL-1β–related CNS inflammation in CUMS rats (Pan et al., 2014).

As a natural dietary bioflavonoid, hesperidin (4′-methoxy-7-O-rutinosyl-3′, 5-dihydroxyflavanone) is mainly distributed in citrus fruits such as fingered citron. Hesperidin produces a variety of pharmacological activities, such as antioxidant, anti-inflammatory, and antiviral effects, together with increasing neurogenesis and promoting memory (Yang et al., 2011). It has been proved that hesperidin could cross the blood-brain barrier (Youdim et al., 2003). Furthermore, studies show that hesperidin has an antidepressant effect (Filho et al., 2013; Donato et al., 2014; Antunes et al., 2016; Li et al., 2016). However, the underlying mechanisms concerning the antidepressant aspect of hesperidin are not fully understood and remain to be explored.

To answer this question, a rat depression model was created using chronic unpredictable mild stress (CUMS), and the antidepressant mechanism of hesperidin inhibits microglia by affecting the NLRP3 signaling pathway was demonstrated.

## Materials and Methods

### Chemicals and Reagents

Hesperidin (S31305-5g) was purchased from Shanghaiyuanye Bio-Technology Co., Ltd (Shanghai, China). Hesperidin (IH0040), Tris-Tricine-SDS-PAGE, BCA Protein Assay Kit, SDS-PAGE loading buffer, 4* (with DTT) and RAPI buffer (high) were provided from Beijing Solarbio Science & Technology Co., Ltd (Beijing, China). Primers of mRNA expressions of NLRP3, caspase-1, ASC glyceraldehyde-3-phosphate dehydrogenase (GAPDH) were obtained by Sangon Biotech Co., Ltd (Shanghai, China). RNAsimple Total RNA Kit DP419, FastKing RT Kit (With gDNase) KR116-02, SuperReal PreMix Plus (SYBR Green) FP205-02 were provided from TIANGEN Biotech (Beijing). Co., Ltd (Beijing, China). IL-βEK301B/3-96, IL-6 EK306/3-24, TNF-αEK382/3-48 ELISA Kit were purchased from MULTI SCIENCES (LIANKE) Biotech Co., Ltd. NLRP3, caspase-1, PYCARD ELISA Kit were provided by Jiyinmei Biological Technology Co., Ltd (Wuhan, China). Rhodamine (TRITC)-Conjugated Rabbit anti-Goat IgG (H + L), Peroxidase-Conjugated Goat anti-Rabbit IgG + L) were purchased from Zhongshan Jinqiao Biological Technology Co., Ltd (Beijing, China). NLRP3 Polyclonal Antibody A5652, caspase-1 Polyclonal Antibody A0964 were provided by ABclonal Biotech Co., Ltd (Wuhan, China) and Rabbit Anti-ASC Polyclonal Antibody bs-6741R were provided by Beijing Biosynthesis Biotechnology Co., Ltd (Beijing, China). AIF-/Iba1 Antibody NB100-1028SS were purchased from Novus Biologicals (Cambridge, UK). Anti-CD68 Antibody (ED1) ab31630 was obtained from Abcam (Cambridge, UK). Coralite488-conjugated Affinipure Goat Anti-Mouse IgG (H + L) was provided from Proteintech Group, Inc (Wuhan, China).

### Animals and Treatments

Male Sprague Dawley rats (170–190 g) were purchased from the SPF (Beijing) Biotechnology Co., Ltd (SCXK (JING) 2016-0002) and used in the present research. The rats were acclimated in a 24.5 ± 0.5°C condition under a 12-h light/dark cycle and freely accessed to food and water. The Ethics Committee for the Welfare of Experimental Animals of Tianjin University of Chinese Medicine approved our animal experiment procedures. After seven days of acclimation, we randomly divided rats into six experimental groups (n = 10): control (No-CUMS), model (CUMS), fluoxetine (20 mg/kg), hesperidin (20 mg/kg), hesperidin (50 mg/kg), and hesperidin (100 mg/kg). Fluoxetine and hesperidin were given orally once daily for 28 consecutive days after the CUMS procedure as follow everyday. In this study, we consulted to a previously described method as the CUMS procedure and made a slight modification (Zhang et al., 2015). According to a “random” schedule, CUMS-induced rats were received to various stressors for 28 days: water or food deprivation for 24 h, swimming in 4°C ice water for 5 min, restraint stress for 2 h, tail nip for 5 min (1 cm from the end of the tail), exposure to white noise for 1 h, soiled cage for 24 h. Rats therefore could not predict the occurrence of stimulation because they were subjected to one of these stressors at different times every day and the same stressor did not come across consecutively over two days.

### Sucrose Preference Test

The animal depression-related symptoms in rodents, especially like anhedonia, are closely related to the sucrose preference test (SPT) (Bolaños et al., 2008). Before the test, rats were transferred to a single cage and need to consume 1% sucrose solution (w/v). Rats with extraordinary sucrose solution consumption were discarded. This test was carried out during the dark phase in accordance with a previous study (Shen et al., 2019), rats were put into a single cage and individually acclimated to consume 1% sucrose solution (w/v) and tap water for 24 h before the CUMS procedure. After adaptation, rats were deprived of water and food for 24 h. Then two new bottles, containing 1% sucrose water and tap water, were given to each rat, respectively. Sucrose solution consumption was calculated for 24 h. Each animal had equal access to the sucrose water and tap water. We recorded the sucrose preference ratio by the amount of (sucrose consumption)/(sucrose consumption + water consumption) × 100%.

### Forced Swimming Test

The depressive-like behavior also was measured by forced swimming test (FST) as described previously (Liu et al., 2016). Briefly, each animal was put into a cylindrical glass container (height, 50 cm; diameter, 20 cm) with 30 cm of water (22 ± 1°C) and forced to swim for 6 min. The time when the mouse stopped struggling and floated with all limbs motionless in the water was considered to be the immobility time. After the test, rats were dried with an electric heater for 10 min.

### Open Field Test

The open ﬁeld test (OFT) is also employed to evaluate the effects of antidepressant treatment (Shyong et al., 2017). The open-field box (50 cm ×50 cm × 30 cm) with the bottom divided into 25 equal squares (10 cm × 10 cm) by black lines and side walls painted black was prepared in advance, rats were placed in the center of the box and could explore the arena for 5 min freely. The video camera was placed on top of the box to record the activity of the rats for 5 min. After each section, feces were removed, and the bottom of the box was disinfected with alcohol. We recorded the quantity of rearings (rat standing on its hind legs) and number of crossings (rat entering into a new sector with four paws) and use these data for statistical analysis.

### Culture of Microglia

Microglia were cultured in Dulbecco’s Modiﬁed Eagle’s medium (DMEM), complemented with 10% fetal bovine serum (FBS) in a humidiﬁed incubator at 37°C ﬁlled with 95% air and 5% CO_2_.

### Quantitative Real-Time Polymerase Chain Reaction (qPCR)

Quantitative real-time polymerase chain reaction (PCR) was performed to measure the relative expression levels of mRNAs. Microglia were cultured into 12-well plates and allowed to adhere for 24 h in DMEM supplemented with 10% FBS. The model group was incubated with culture medium containing 100 ng/ml lipopolysaccharide (LPS) for 3 h (Scheiblich et al., 2017). The hesperidin group was primed 3 h prior to experiments with 100 ng/ml LPS before cells were treated with hesperidin (20, 40, 80 μmol/L). Cultured cells were collected, the PFC was homogenized, and a Total RNA kit was used to prepared the total RNA according to the manufacturer’s instructions. Total RNA concentration was detected using a K5500 Micro-Spectrophotometer (VWR, USA). CDNAs, used as templates for real-time PCR were then generated using the FastKing RT Kit (with gDNase), following the manufacturer’s protocol. The qPCR amplification conditions were as follows: 95°C for 10 min, followed by 40 cycles of 95°C for 10 s and 60°C for 30 s using IQ5 Multicolor Real-Time PCR Detection System (BIO-RAD, USA). The primer sequences were obtained as required, and were as follows **Table 1**. The 2^−ΔΔ^Cq method was applied for relative gene expression quantification (Livak and Schmittgen, 2002).

**Table 1:** Primer sequences.

Gene	Forward primer (5′-3′)	Reverse primer (5′-3′)
NLRP3	CTCGCATTGGTTCTGAGCTC	AGTAAGGCCGGAATTCACCA
Caspase-1	ACAAAGAAGGTGGCGCATTT	GTGCTGCAGATAATGAGGGC
ASC	TGTGCTTAGAGACATGGGCA	ACTGCCTGGTACTGTCCTTC
GAPDH	GACAACTTTGGCATCGTGGA	ATGCAGGGATGATGTTCTGG

### Western Blotting Analysis

The PFC samples were put into ice-cold RIPA buﬀer solution for homogenate. After centrifugation, we used a BCA kit to measure the protein concentration of the lysates. After denaturation, proteins samples were separated using electrophoresis on SDS-PAGE gels about 40 min and then transferred onto PVDF membranes. Protein bands were blocked in Tris-buffered saline-tween 20 (TBST) solution with 3% (w/v) skimmed milk under gentle rocking for 1 h at room temperature (RT) and incubated with 1:1000 dilution of primary antibodies (NLRP3, caspase-1, ASC) at 4°C. Then membranes were washed three times using TBST and probed with 1:10,000 dilution of HRP-conjugated secondary antibody for 2 h at RT. Immunoblots were visualized using ECL reagents (VILBER LOURMAT, France) and band densities were quantified using Image J analyzer software. β-actin was used as normalization control. Each experiment was repeated at least three times.

### Immunofluorescence Staining

Methods use OTC to make frozen sections of rat brain tissue. After blocking with 10% goat serum, the slides were incubated with AIF-/Iba1 antibody overnight. The sections were washed with PBS and rhodamine (TRITC)-conjugated rabbit anti-goat IgG (H + L) antibody was used as a secondary antibody, followed by the addition of DAPI. Finally, the confocal microscope (OLYMPUS, Japan) was used to observe the iba1-positive cells in the PFC.

### Flow Cytometry Assay

Microglia were cultured into 12-well plates and allowed to adhere for 24 h in DMEM supplemented with 10% FBS. The model group was incubated with culture medium containing 100 ng/ml lipopolysaccharide (LPS) for 3 h. The hesperidin group was primed for 3 h prior to experiments with 100 ng/ml LPS before cells were treated with hesperidin (20, 40, 80 μmol/L). Then, each well was trypsinized, collected, washed with phosphate buffer saline (PBS) and incubated with Anti-CD68 ANTIBODY (ED1) and Coralite488-conjugated Affinipure Goat Anti-Mouse according to the manufacturer’s instructions. Activated microglia was measured by flow cytometry (Millipore, France) and the data were analyzed using guavaSoft 3.1.1.

### Enzyme-Linked Immunosorbent Assay

The inflammasome components (NLRP3, ASC, and pro-caspase-1) have been released into the microglia supernatant after the NLRP3 inflammasome was activated (Baroja-Mazo et al., 2014). According to the manufacturer’s instructions, the expression levels of pro-inflammatory mediators (IL-1β, IL-6, and TNF-α) and NLRP3 inflammasome assembly (NLRP3, caspase-1, ASC) in the PFC of CUMS-induced rats and cell supernatant of LPS-induced microglia were measured using commercial ELISA kits. The microplate reader (Thermo, USA) was to determine the optical density (OD) value at 450 nm.

### Statistical Analysis

Data were presented as mean ± S.D. Statistical analyses were performed using One-Way ANOVA with SPSS 16.0 software for Windows. Student’s *t*-test was used to compare two groups, and P<0.05 was considered as statistically significant. ^##^P<0.01, ^#^P<0.05, **P<0.01, *P<0.05.

## Results

### Hesperidin Attenuates CUMS-Induced Depressive-Like Behavior in Rats

#### Effects of Hesperidin on SPT

**Figure 1:**
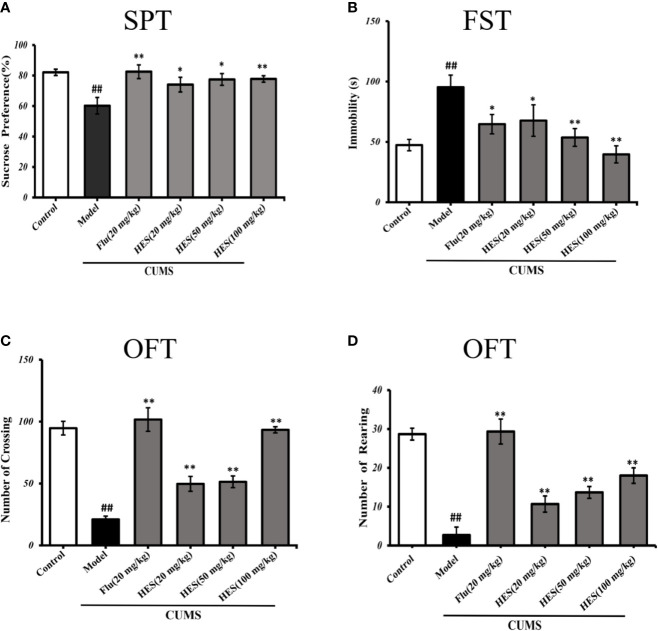
Effects of fluoxetine and hesperidin treatments on depression-like behavior in the sucrose preference test **(A)**, forced swim test **(B)**, and open ﬁeld test **(C, D)**. The results are shown as mean ± S.D. (n = 8). ^##^P < 0.01 vs. the Control group, **P < 0.01 and *P < 0.05 vs. the model chronic unpredictable mild stress (CUMS) group.

As shown in **Figure 1A**, the results of the SPT were summarized to further investigate the antidepressant effects of hesperidin. After a four-week period of CUMS procedure, the amount of sucrose solution consumption in model group was significantly reduced compared with the control group in rats (P<0.05 or P<0.01, **Figure 1A**). Four-week treatment of hesperidin and fluoxetine significantly increased the amount of sucrose solution consumption compared with the model group. These results imply that hesperidin could improve the anhedonia-like state in the SPT.

#### Effects of Hesperidin on FST

The immobility time in the FST was measured to further illustrate the effect of hesperidin on depression. Compared with the controls, data analysis indicated that CUMS-induced rats manifested a significantly longer immobility time in FST (P<0.05 or P<0.01, **Figure 1B**). Treatment of hesperidin (20, 50, and 100 mg/kg) and fluoxetine (20 mg/kg) significantly ameliorated the depressant-like states induced by the CUMS model in the FST.

#### Effects of Hesperidin on OFT

The OFT includes the numbers of crossings and rearings which were known to reflect the locomotor activity of animals, the fewer the number of squares visited in the center of the open-field box reflects a more depressive-like state. The results revealed that the numbers of crossings and rearings were significantly lower in the model group compared with the control group (P<0.05 or P<0.01, **Figures 1C, D**). The reduction of locomotor activity as evidenced by a decreased crossings and rearings number was relieved by treatment with hesperidin (20, 50, and 100 mg/kg) and fluoxetine (20 mg/kg).

### Hesperidin Inhibits the Activation of Microglia in Rats

**Figure 2:**
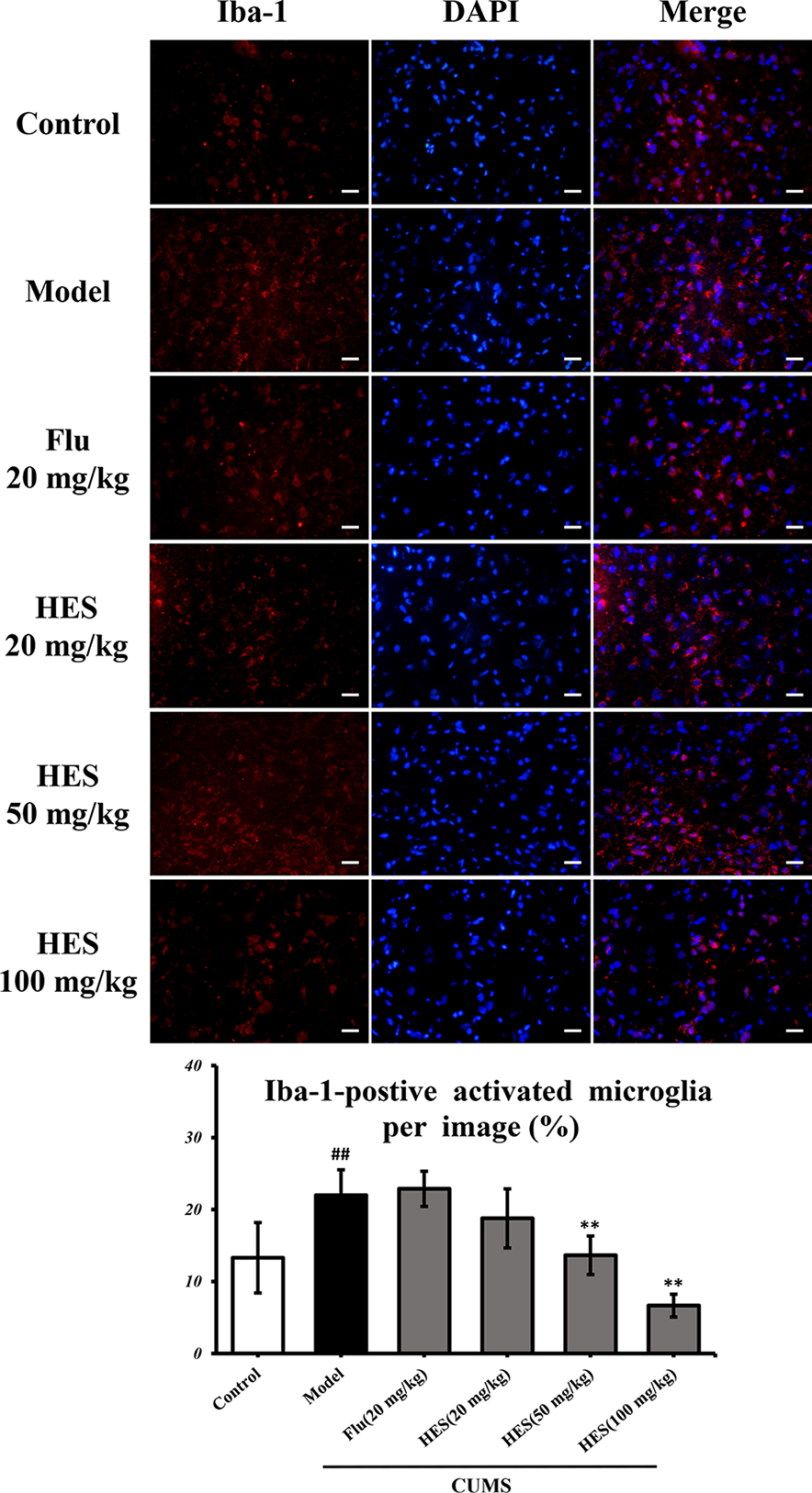
Effects of fluoxetine and hesperidin on the PFC iba-1–positive cells in CUMS-induced rats. The results are shown as mean ± S.D. (n = 3). ^##^P < 0.01 vs. the Control group, **P < 0.01 vs. the model chronic unpredictable mild stress (CUMS) group. Scale bars = 200 μm.

Depression is closely related to the generation of microglia-derived factors. As shown in **Figure 2**, CUMS-treated rats had significantly increased Iba-1–positive cells in the PFC (P<0.05 or P<0.01). One-way ANOVA indicated that the effect of treatment was significant. Analysis indicated that hesperidin (20, 50, and 100 mg/kg) as well as fluoxetine (20 mg/kg) prevented the elevation of Iba-1–positive cells induced by CUMS.

### Effects of Hesperidin on the mRNA and Protein Expression of NLRP3 Signaling Pathway in the PFC

**Figure 3:**
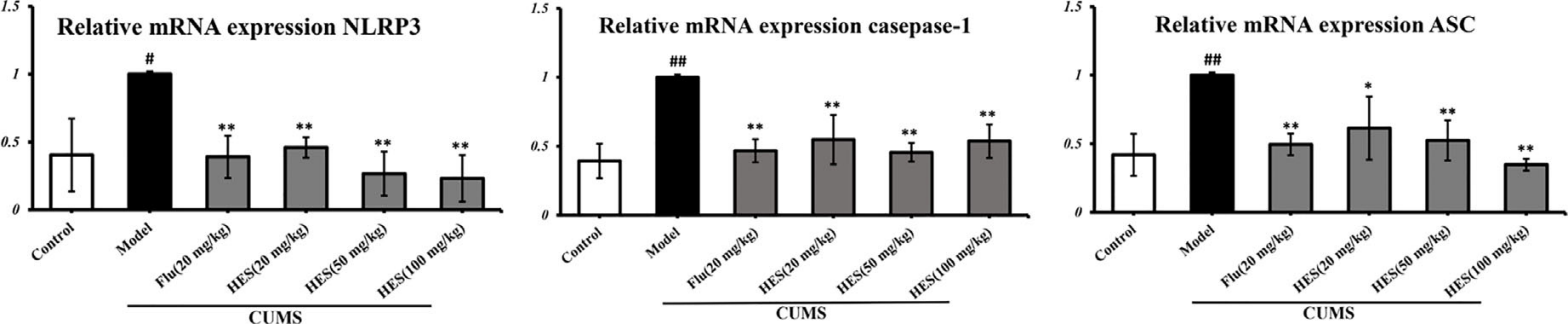
Effects of fluoxetine and hesperidin on NLRP3 signaling pathway in the PFC of CUMS-induced rats. Total mRNA was harvested and mRNA expression levels of NLRP3, caspase-1, ASC were measured by quantitative real-time PCR. The results are shown as mean ± S.D. (n = 3). ^##^P < 0.01 and ^#^P < 0.05 vs. the Control group, **P < 0.01 and *P < 0.05 vs. the model chronic unpredictable mild stress (CUMS) group.

**Figure 4:**
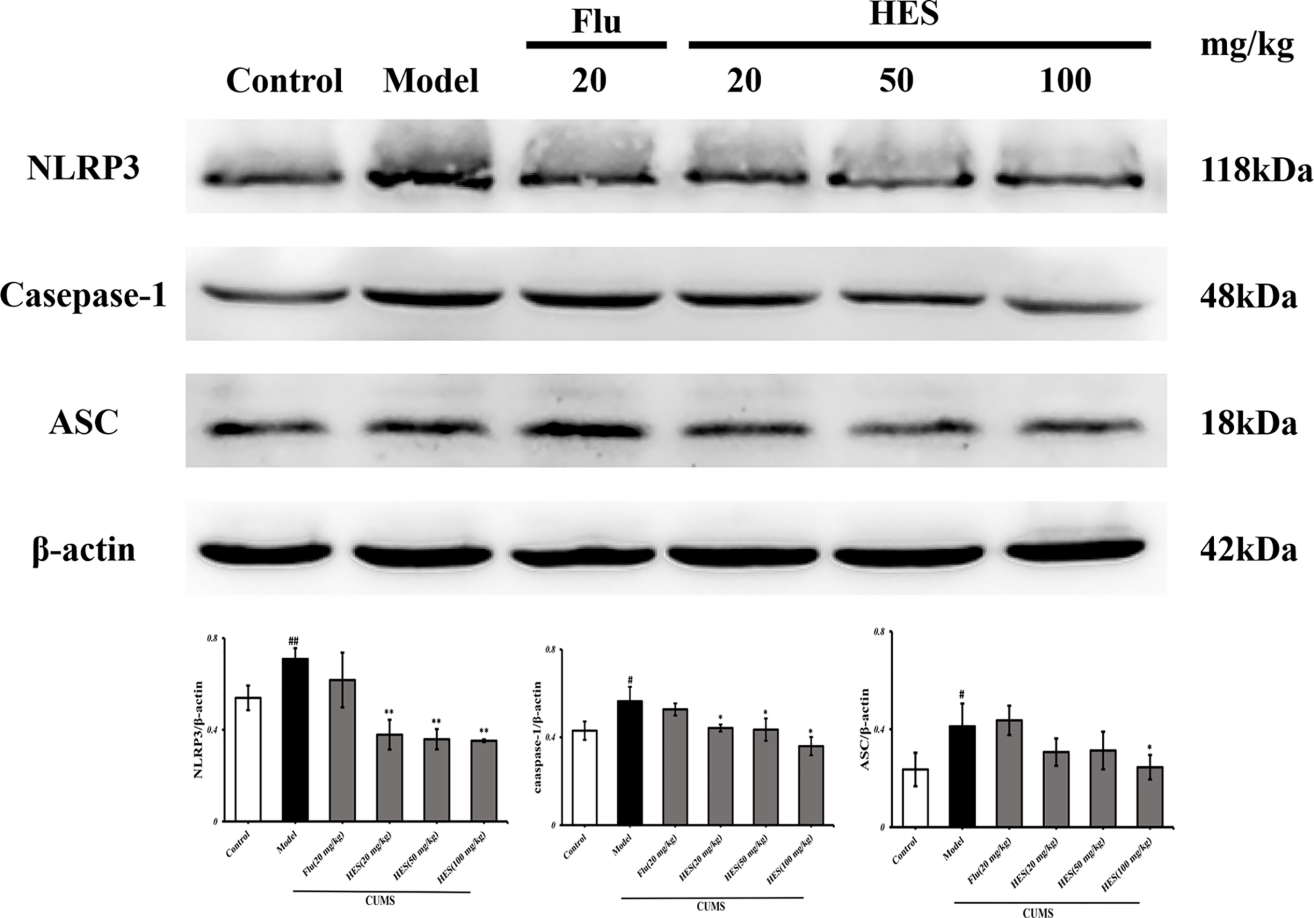
Effects of fluoxetine and hesperidin on NLRP3 signaling pathway in the PFC of CUMS-induced rats. The protein levels of NLRP3, caspase-1, ASC were determined by Western blot analysis. The results are shown as mean ± S.D. (n = 3). ^##^P<0.01 and ^#^P<0.05 vs. the Control group, **P<0.01 and *P<0.05 vs. the model chronic unpredictable mild stress (CUMS) group.

We used PCR and Western blot to monitor the differences of NLRP3 inflammasome activation in the level of genes and proteins in the PFC of CUMS rats (P<0.05 or P<0.01, **Figures 3** and **4**). As described in **Figures 3** and **4**, we observed that the expression of NLRP3 inflammasome components (NLRP3, caspase-1, and ASC) were significantly transferred to a higher post at genes and protein levels compared with the control group, whereas fluoxetine (20 mg/kg) and hesperidin (20, 50, and 100 mg/kg) treatments resulted in decreased NLRP3, caspase-1, and ASC compared with the model group in the PFC.

### Effects of Hesperidin on the Levels of Pro-Inflammatory Cytokines In Pfc

**Figure 5:**
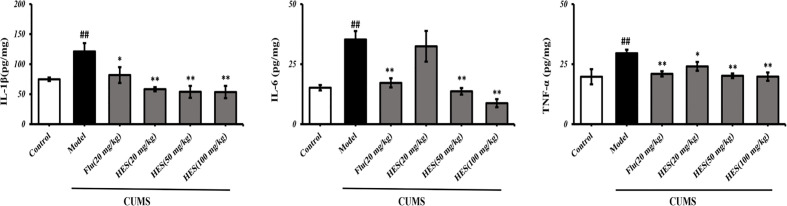
Effects of fluoxetine and hesperidin on pro-inflammatory cytokines in the PFC of CUMS-induced rats. The results are shown as mean ± S.D. (n = 3). ^##^P < 0.01 vs. the Control group, **P < 0.01 and *P < 0.05 vs. the model chronic unpredictable mild stress (CUMS) group.

To confirm hesperidin’s effect on inflammation, we used ELISA kits to measure the levels of pro-inflammatory cytokines in the PFC of rats. The results show in **Figure 5**, CUMS significantly increased the IL-1β, IL-6, TNF-α expression in the PFC (P<0.05 or P<0.01). In addition, CUMS-stimulated expression of IL-1β, IL-6, TNF-α was clearly reduced by hesperidin (20, 50, and 100 mg/kg) and fluoxetine (20 mg/kg).

### Hesperidin Inhibits the Activation of Microglia

**Figure 6:**
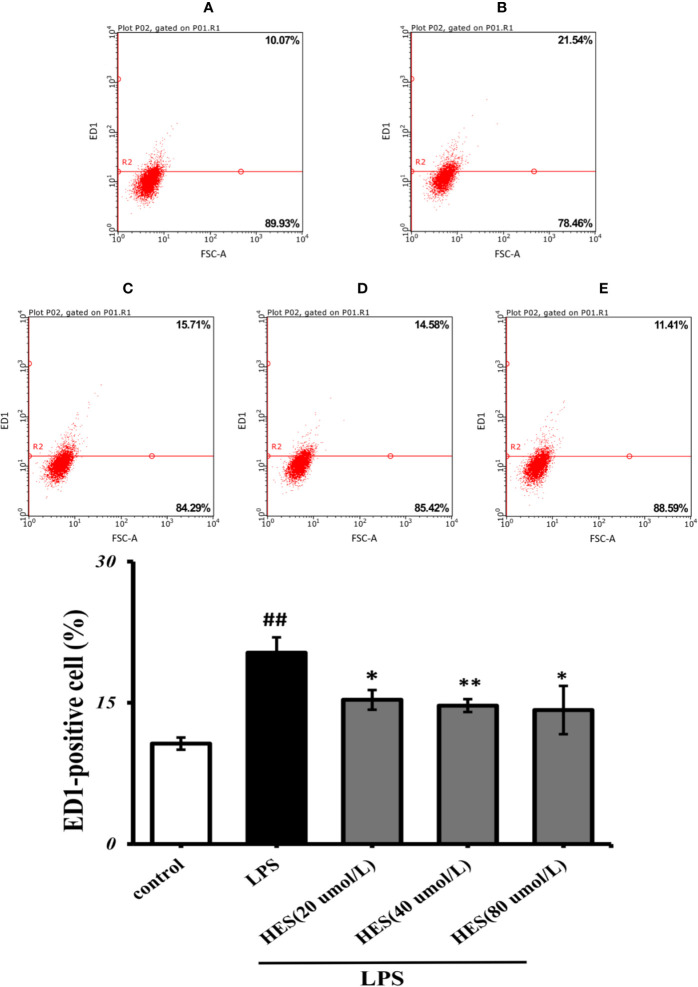
Effects of hesperidin on LPS-induced activation in microglia. **(A)** Control, **(B)** LPS **(C)** HES (20 μmol/L) + LPS, **(D)** HES (40 μmol/L) + LPS, **(E)** HES (80 μmol/L) + LPS. The results are shown as mean ± S.D. (n = 3). ^##^P<0.01 vs. the Control group, **P < 0.01 vs and *P < 0.05. LPS group.

Flow cytometry analysis demonstrated that LPS-induced activation in microglia was significantly promoted (P<0.05 or P<0.01, **Figure 6**). At the same time, the effects of activation in LPS-treated cells were further diminished by hesperidin.

### Effects of Hesperidin on the NLRP3 Signaling Pathway in Microglia

**Figure 7:**
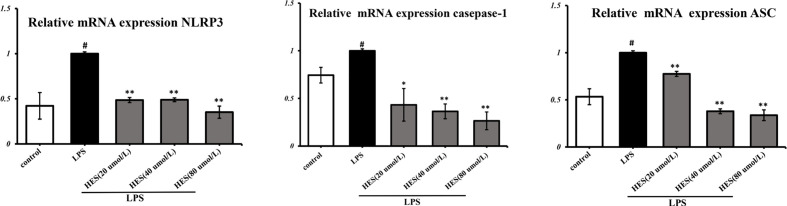
Effects of hesperidin on NLRP3 signaling pathway mRNA expression in microglia. The results are shown as mean ± S.D. (n = 3). ^#^P < 0.05 vs. the Control group, **P < 0.01 and *P < 0.05 vs. LPS group.

**Figure 8:**
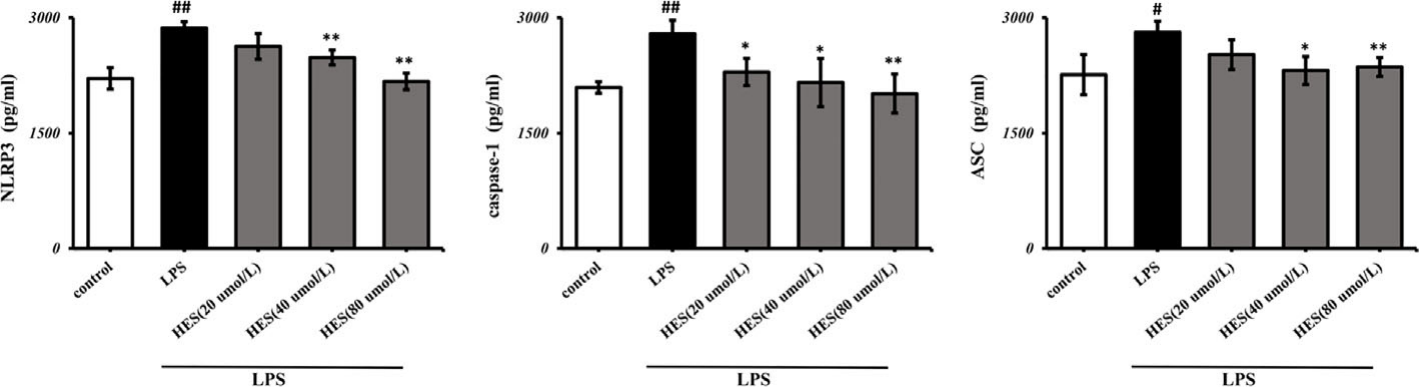
Effects of hesperidin on NLRP3 signaling pathway in microglia. The results are shown as mean ± S.D. (n = 3). ^##^P < 0.01 and ^#^P <0.05 vs. the Control group; **P < 0.01 and *P <0.05 vs. LPS group.

To detect the effects of hesperidin on LPS-induced microglia in the intracellular pathway, the NLRP3 signaling pathway was tested. We performed PCR and ELISA analysis of NLRP3 signaling in the microglia. Our data show that intensity of NLRP3, caspase-1, ASC signal was dramatically increased in the LPS-induced model (P < 0.05 or P < 0.01, **Figures 7** and **8**). Meanwhile, hesperidin significantly decreased the intensity of NLRP3, caspase-1, ASC signal in microglia, indicating its prohibitive effects to microglia.

### Effects of Hesperidin on Pro-Inflammatory Cytokines in Microglia

**Figure 9:**
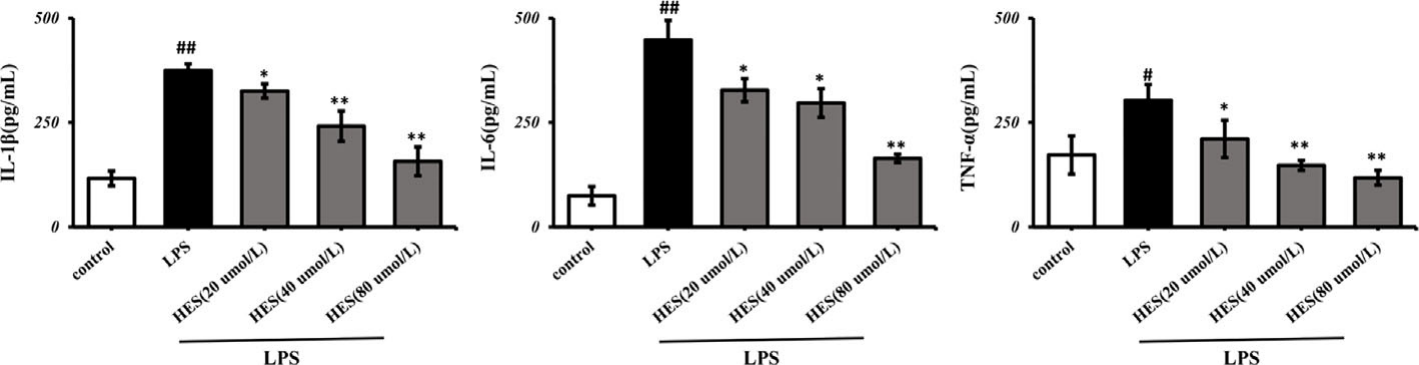
Effects of hesperidin on pro-inflammatory cytokines in the supernatant of microglia. The results are shown as mean ± S.D. (n = 3). ^##^P < 0.01 and ^#^P < 0.05 vs. the Control group, **P < 0.01 and *P < 0.05 vs. LPS group.

In **Figure 9**, the IL-1β, IL-6, TNF-α levels significantly increased in the LPS-induced group (P < 0.05 or P < 0.01). Further analysis indicated that hesperidin, as well as fluoxetine, prevented the elevation of IL-1β, IL-6, TNF-α levels induced by LPS.

These results indicated that hesperidin may exert key antidepressant-like effects against CUMS-induced depression in rats *via* regulating the NLRP3 signaling pathways.

## Discussion

Conventional western medicine treatment of depression mainly includes tricyclic antidepressants (TCA), selective 5-HT reuptake inhibitors (SSRIs), 5-HT and NE reuptake inhibitors (SNRI), 5-HT receptor antagonist and 5-HT reuptake inhibitors (SARI), monoamine oxidase inhibitors (MAOI) and norepinephrine reuptake inhibitors (NRI), etc., which can cause various side effects, such as headache, dry mouth, constipation, vomit, insomnia, and anxiety. Hence, we need to explore novel treatment for depression. Many studies indicate that traditional Chinese medicines are effective in treating depression. Acorus tatarinowii decoction has strong anti-depressive effects on the CUMS rats by inhibiting the transcription and expression of nuclear transcription factor NF-κb (Wu et al., 2012). By inducing the expression of cortical pro-inflammatory cytokines IL-1 and TNF-α, extract of radix paeoniae alba reduced the level of inflammatory cytokines in the rat brain, thereby improving depressive symptoms in the CUMS rats (Wang and Chen, 2013). A lot of studies also prove that hesperidin has an effect on depression (Antunes et al., 2016; Morteza et al., 2018; Morteza et al., 2018; Fu et al., 2019).

Behavioral experiments are often used to evaluate whether drugs have antidepressant properties, and sucrose preference, forced swimming and open-field experiments can be used as indicators to evaluate depression. In the OFT, CUMS further decreased the amount of rearings and crossings, suggesting a loss of interest to explore surroundings. This study demonstrates that hesperidin can effectively alleviate CUMS-induced depression-like behavior in rats and improve their behavioral defects. Especially the rats in the hesperidin (100 mg/kg) group showed the most obvious differences compared with the rats in the model group. As the effect and complex mechanism of hesperidin’s anti-depression remains unclear, so we need to identified the mechanism responsible for hesperidin’s effect on depression in CUMS rats. Within recent decades, there are increasing number of studies shown that depression or other anxiety disorders might be leaded by inflammation agents such as LPS in adulthood. We found that the kappa B (NF-κb) inflammatory pathway could be significantly activated by CUMS procedure in rats. The NLRP3 inflammasome, composed of NLRP3, caspase-1, and apoptosis-associated speck-like protein containing a caspase recruitment domain (ASC), is regarded as an essential mediator of IL-1β function (Haneklaus et al., 2013). The activation of caspase-1 which mediated by the NLRP3 inflammasome lead pro-IL-1β to form mature IL-1β in PFC of CUMS rats (Green and Nolan, 2014; Musaelyan et al., 2014; Pan et al., 2014; Scheiblich et al., 2017). The CUMS-induced chronic depression-related symptoms could be impeded by the NLRP3 signaling pathway inhibitors Ac-YVAD-CMK and VX-765 significantly (Zhang et al., 2015). It has been proved that the NLRP3 inflammasome is involved in animal depressive-like behaviors in first time and published in CNS Neuroscience and Therapeutics (Zhang et al., 2014). Further research showed that the expression of NLRP3 increased in blood cells from patients with major depressive disorder and the NLRP3 inflammasome may be a new target in major depressive disorder (Alcocer-Góomez and Cordero, 2014; Alcocer-Gómez et al., 2014). Based on these etiological findings, it has been suggested that anti-inflammatory treatment might yield antidepressant properties (Tyring et al., 2006; Zhang et al., 2014). A plenty of research manifest that hesperidin have anti-inflammatory effect on depression and other disorders (Adeniyi et al., 2017; Fu et al., 2019; Heo et al., 2019; Selim et al., 2019; Sun Hyo et al., 2019).

In this study, hesperidin was found to reduce the expression of NLRP3 inflammasome (NLRP3, caspase-1, and ASC) activation in the PFC of CUMS-induced rats and microglia. Further study shows that knockout of NLRP3 gene inhibits the activation of NF-κb protein complex in the CUMS rats. These data prove that NLRP3 inflammasome mediates depression-like symptoms in CUMS rats (Su et al., 2017). Our data also showed that hesperidin decreased the number of activated microglia and the level of pro-inflammatory cytokines (IL-1β, IL-6, and TNF-α) in the PFC. At the same time, hesperidin inhibited LPS-induced the activation of pro-inflammatory cytokines and the NLRP3 signaling pathway in microglia, especially the group of hesperidin (80 μmol/L). The up-regulated levels of pro-inflammatory cytokines released by activated microglia results in neuroinflammatory responses which have been found to increase the risk of neuropsychiatric symptoms, such as depression in many research (Wong et al., 2008; Baune et al., 2010; Dahl et al., 2014; Du et al., 2019). In addition, IL-6 is involved in neurogenesis, neural mediation, and TNF-α plays a major role in the brain’s immune response and is an important indicators for depression (Cai et al., 2018). Huiling Fu et al. also showed hesperidin have anti-inflammatory effect on depression by the HMGB1/RAGE/NF-κb and BDNF/TrkB pathways. Although we both pay attention to the inflammation and depression, this article highlights the microglia and NLRP3 inflammasome *in vivo* and vitro experiments.

In the present study, hesperidin was confirmed to have anti-depressive effects in CUMS-induced rats. Its potential pharmacological mechanisms may be included the inhibition of NLRP3 inflammasome and microglia activation in the PFC of CUMS rats.

In summary, the results in the present study demonstrate the experimental evidence that hesperidin might be a promising strategy to alleviate depression in the future.

## Data Availability Statement

The raw data supporting the conclusions of this article will be made available by the authors, without undue reservation.

## Ethics Statement

The animal study was reviewed and approved by the Welfare of Experimental Animals of Tianjin University of Chinese Medicine.

## Author Contributions

YB conceived and designed the experiments. LX and ZG wrote the paper and performed the experiments. LX, HL, and BJ analyzed the data. LX, RS, and YW wrote the protocol. ZZ and MC helpful revision on the text and grammar. All authors contributed to the article and approved the submitted version.

## Funding

This work was by supported by the National Key R&D Program of China (2018YFC1706500), Technical System Upgrading and Product Development of the Whole Industry Chain of Glycyrrhiza Uralensis. The Anti-Depressive Effects of Hesperidin and the Relative Mechanisms Based on the NLRP3 Inflammatory Signaling Pathway (YJSKC-20191038).

## Conflict of Interest

The authors declare that the research was conducted in the absence of any commercial or financial relationships that could be construed as a potential conflict of interest.
